# Effects of multiple pro-inflammatory stimuli *in utero* on the ileum of extremely premature ovine fetuses

**DOI:** 10.3389/fimmu.2023.1150208

**Published:** 2023-05-19

**Authors:** Julia Heiter, Matthew W. Kemp, Owen B. Spiller, Dominique Singer, John P. Newnham, Suhas G. Kallapur, Alan H. Jobe, Boris W. Kramer

**Affiliations:** ^1^ Division of Mental Health and Neuroscience, Maastricht University Medical Center, Maastricht, Netherlands; ^2^ Division of Neonatology and Pediatric Critical Care Medicine, University Medical Center Eppendorf, Hamburg, Germany; ^3^ Department of Obstetrics and Gynaecology, Yong Loo Lin School of Medicine, National University of Singapore, Singapore, Singapore; ^4^ Division of Infection and Immunity, University Hospital of Wales, Cardiff, United Kingdom; ^5^ University of Western Australia, King Edward’s Memorial Hospital, Crawley, WA, Australia; ^6^ Division of Neonatology and Developmental Biology at University of California, Los Angeles (UCLA) Health, Mattel Children’s Hospital, Los Angeles, CA, United States; ^7^ University of Cincinnati, Cincinnati Children’s Hospital, Cincinnati, OH, United States

**Keywords:** chorioamnionitis, enteric nervous system, preterm fetal ileum, intestinal inflammation, necrotizing enterocolitis

## Abstract

**Introduction:**

Chorioamnionitis is common in preterm birth and associated with a higher risk of intestinal inflammation and necrotizing enterocolitis. The intestinal inflammation influences the enteric nervous system development. We hypothesized that inflammation and innervation in the fetal ileum may be modified by chorioamnionitis induced by repeated challenge with lipopolysaccharide and/or preexisting *Ureaplasma parvum* infection at very low gestational age equivalent to 60% of term.

**Materials and methods:**

Time mated ovine fetuses were exposed by intraamniotic injections to chronic *Ureaplasma parvum* for 24 days and/or lipopolysaccharide for 7 days, 2 days, or 7 & 2 days before delivery at 94 +/-2 days of gestational age (term at approximately 150 days). Intestinal inflammation as well as structural changes of the enteric nervous system were assessed.

**Results:**

Lipopolysaccharide exposure increased CD3 and myeloperoxidase-positive cells (p < 0.05). Repetitive exposure to lipopolysaccharide or combined *Ureaplasma parvum* & lipopolysaccharide exposure increased intestinal inflammation (p < 0.05). The reduction of nuclei of neurons was most significant with repetitive lipopolysaccharide exposures but could be detected in all other intervention groups compared to the control group. Astrocyte-like glial cells increased if exposure to lipopolysaccharide was only 2 days before delivery or chronic exposure to *Ureaplasma parvum* existed beforehand (p < 0.05).

**Discussion:**

After exposure to chorioamnionitis induced by *Ureaplasma parvum* and/or lipopolysaccharide, inflammatory responses as well as structural changes of the enteric nervous system were more pronounced the longer and the more frequent the exposure to pro-inflammatory stimuli before birth. These changes may cause functional effects of clinical importance.

## 1 Introduction

Premature delivery is frequently associated with intrauterine infection (1–4). Chorioamnionitis, an inflammation of the inner (amnion) and outer (chorion) fetal membranes, and funisitis, an inflammation of the umbilical cord, is commonly caused by ascending infections into the uterine cavity (1, 5). While numerous bacterial species have been detected in the amniotic fluid, the most frequently identified species are genital mycoplasmas, *Ureaplasma species*, *Gardnerella vaginalis*, and *Fusobacteria* spp (1, 5). E. coli also belongs to one of the pathogens associated with chorioamnionitis (1). In up to two-thirds of all cases, chorioamnionitis is associated with multiple pathogens (6). The subsequent microbial growth exposes the fetus to multiple bacterial toxins or products of inflammation that may cause a fetal inflammatory response syndrome (FIRS), for example, lipopolysaccharide (LPS) derived from E. coli activates the Interleukin-1 pathway (7). FIRS is associated with postnatal adverse outcomes in multiple fetal organs including the gastrointestinal tract (8–10). As the gestational age of premature delivery decreases, the frequency and severity of complications increase (2, 11).

Preterm infants, especially those < 28 weeks of gestation, exposed to chorioamnionitis can have a wide spectrum of adverse intestinal consequences ranging from poor nutritional uptake and postnatal growth deficits to life-threatening necrotizing enterocolitis (NEC) (9, 10). In addition, former NEC patients may have lifelong consequences beyond the directly affected gut (12). Patients who survive after NEC may have lower pain thresholds later in life (13). This association is poorly understood. Many gut related pathologies result from the enteric nervous system (ENS), which consists of more than 500 million neurons and glia cells within the wall of the bowel and regulates most aspects of gastrointestinal function (14, 15). The ENS innervates smooth muscles cells, secretory epithelial cells as well as the blood vessels within the intestinal wall. Hence, the ENS is responsible for regulating gastrointestinal tract movement, gastric acid secretion, microbiome composition, fluid movement across the lining epithelium, local blood flow, nutrient handling and interaction with the immune and endocrine systems of the gut (14, 16). In a preclinical model of chorioamnionitis, we have shown at 80% of term gestation that chronic exposure to *Ureaplasma parvum* (UP) induced chorioamnionitis is associated with injury in the enteral nervous system (17).

Intra-amniotic exposure to either lipopolysaccharide (LPS), UP or other pro-inflammatory mediators induces intestinal inflammation and mucosal injury in fetal sheep (18–20). In more mature fetal sheep at 120-123 days of gestational age, a cross tolerance to several toll like receptor signals in blood and lung inflammatory cells after repetitive LPS injections into the amnionitic fluid has been observed (21, 22). The importance of this cross tolerance is unclear but may limit the injury induced by inflammation. However, structural changes of the ENS after exposure to pro-inflammatory stimuli have not been evaluated at extremely low gestational age of 60% of term gestation, corresponding to approximately 24 weeks of gestation in the human, thus referring to extreme prematurity defined as < 28 weeks of gestation in humans (11). We hypothesized that there will be changes in the ENS because repetitive pro-inflammatory stimuli affect the fetal immune system, inducing systemic changes in the corresponding immune cells at extremely low gestational ages (21, 23). Inflammation and innervation both affect ileal development. However, tolerance effects on the extremely premature fetal ileum have not yet been studied. Furthermore, LPS responsiveness is profoundly decreased after chronic UP exposure to the fetal lung and gut (17, 24). It remains unclear, whether these tolerance mechanisms occur in the intestinal immune system at 60% of term gestation.

We hypothesized that inflammation and innervation in the extremely premature fetal ovine ileum may be affected by repetitive injections of LPS and/or preexisting UP chorioamnionitis.

## 2 Material and methods

### 2.1 Animals

All animal experiments were performed at the University of Western Australia, Subiaco, Western Australia, Australia, with approval of the Animal Ethics Committee (reference number RA/3/100/312). The study was carried out in accordance with the “Animal Welfare Act (2002)” and the requirements of the Australian code for care and use of animals for scientific purposes (8^th^ edition, 2013).

### 2.2 Experimental design

**Figure 1:**
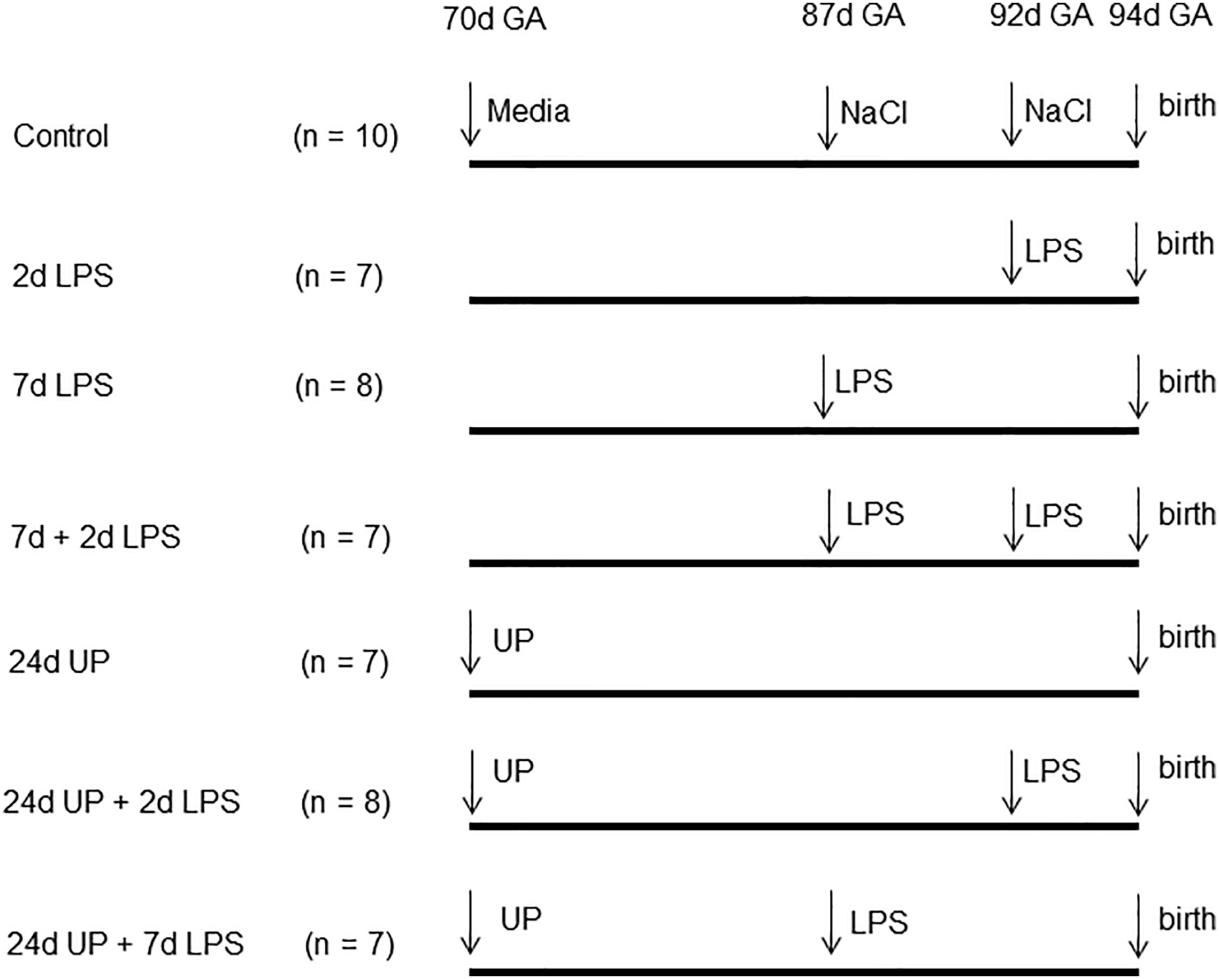
Experimental design: Pregnant ewes received either LPS 2, 7 or 7 and 2 days before delivery or only UP 24 days before delivery or UP 24 days before delivery and LPS either 2 or 7 days before delivery. The control group received the equivalent amount of media or saline. Fetuses were delivered at 94 days gestational age (GA).

54 time-mated Merino ewes with singleton fetuses were randomly assigned to one of the six treatment groups of seven to eight animals each or a 10 animal combined control group (**Figure 1**). Depending on the study group the ewes received ultrasound guided intra-amniotic injections of either 2 mL saline 2 or 7 days before delivery (thus at 92 or 87 days of gestation) or 2 mL culture media 24 days before delivery (thus at 70 days of gestation) - combined control group - or one or two of multiple pro-inflammatory stimuli: 10 mg LPS (Escherchia coli 055:B5; Sigma-Aldrich, St. Louis, MO, USA), 7 days (thus at 87 days of gestation), 2 days (thus at 92 days of gestation), or 7 & 2 days (thus at 87 days and 92 days of gestation), before delivery (7d LPS group, 2d LPS group, 7d + 2d LPS group), 2x 10^7^ CCU (colour-changing units) of *Ureaplasma parvum* serovar 3 (strain HPA5) at 24 days (thus at 70 days of gestation), before delivery (24d UP group), or combinations thereof: 2x 10^7^ CCU of UP, 24 days before delivery plus 10 mg LPS, at 2 or 7 days before delivery (24d UP + 2d LPS group, 24d UP + 7d LPS group). Doses and time points were used as evaluated beforehand (25). Fetal terminal ileum samples were collected under sterile conditions during autopsy after Caesarean section under terminal anaesthetic conditions (intravenous pentobarbital) at 94+/-2 days of gestational age (term 150 days) (26, 27). Fetal samples were processed for frozen section (snap-frozen in liquid Nitrogen) as well as fixed in formalin (48 hours) prior to paraffin embedding.

### 2.3 Cell-type identifications

Specific cell types were identified using the following markers: CD3 positive T-lymphocytes (20, 28, 29) for the acquired immune system, myeloperoxidase (MPO) for monocytes and neutrophils (9, 18, 30) for the innate immune system. The cells were evaluated in the mucosa and submucosal tissue of the fetal ileum. ENS was identified by synaptophysin staining (31, 32) to analyze synaptic activity in the myenteric plexus. We also stained for serotonin in enterochromaffin cells (32) in the mucosa of the fetal ileum, neuronal nuclei (NeuN) (32, 33), neuronal growth factor receptor (NGFR) (34) and vimentin for astrocyte like glial cells (35, 36) all in the myenteric plexus, as a representative for the entire ENS, of the fetal ileum.

### 2.4 Antibodies

The following antibodies were used as primary antibodies: Dako polyclonal rabbit anti-human CD3, cat#: A0452, concentration 1:1000 (Glostrup, Denmark), Dako polyclonal rabbit anti-human MPO, cat#: E0398, concentration 1:1000 (Glostrup, Denmark), Millipore monoclonal mouse anti-synaptophysin, cat#: MAB5258, concentration 1:16000 (Billerica, MA, USA), Maastricht University polyclonal rabbit anti-serotonin, concentration 1:16000 (Maastricht, The Netherlands), Millipore monoclonal mouse anti-NeuN, cat#: MAB377, concentration 1:800 (Billerica, MA, USA), Thermo Fisher Scientific monoclonal mouse anti-human NGFR, cat#: MA1-18418, concentration 1:2000 (Waltham, MA, USA); which was a generous gift of C. Heijmans and W. van Gemeert MUMC+, Maastricht, The Netherlands, Cell Signalling monoclonal rabbit anti-vimentin, cat#: 5741S, concentration 1:800 (Danvers, MA, USA). The following secondary antibodies were used: Dako biotin labelled polyclonal swine anti-rabbit, cat#: E0353, concentration 1:200 (Glostrup, Denmark) and Dako biotin labelled polyclonal goat anti-mouse, cat#: E0433, concentration 1:200 (Glostrup, Denmark).

### 2.5 Immunohistochemistry

Formalin-fixed terminal ileum was embedded in paraffin and 4 µm sections were cut. After deparaffinization and rehydration endogenous peroxidase activity was blocked with 0,3% H_2_O_2_ diluted in tris-buffered saline (TBS), followed by antigen retrieval with 10 mM citric acid which was not necessary for MPO staining. To block non-specific binding 1% bovine serum albumin (Sigma) was used for 30 minutes for MPO-, serotonin- and vimentin-staining. All other stainings did not need blockage of non-specific binding. Thereafter, sections were incubated with the primary antibody over-night at room temperature. After washing the next morning, slides were incubated with the secondary antibody for 1 hour, followed by washing and incubation of avidin/biotin complex for 30 minutes. Immunoreactivity was detected with nickel sulfate-diaminobenzidine (NiDAB) from Dako (Glostrup, Denmark). After counterstaining with 0,1% Nuclear Fast Red (Sigma-Aldrich, St. Louis, MO, USA), sections were washed, dehydrated and coverslips mounted.

Evaluations were performed with digital pictures taken with Micromanager MM studio version 1.4.20 (Vale Lab, University of California, San Francisco, USA) by light microscopy with an Olympus BX5 (Shinjuku, Tokyo, Japan). CD3 and MPO expressing cells were counted in mucosa and submucosal tissue in three non-overlapping high-power fields (magnification x 200) and the average numbers of positively stained cells per high-power field per animal are presented. Similarly, enterochromaffin cells (serotonin positive cells) were counted in the mucosa and neuronal nuclei were counted in ganglia of the myenteric plexus. As for synaptophysin, serotonin, NGFR and vimentin positive areas, ImageJ version 1.51 (Laboratory for Optical and Computational Instrumentation, University of Wisconsin-Madison, Madison, WI, USA) was used to calculate the area fraction (%) of positive stained areas in the myenteric plexus in relation to the total area underneath the epithelial layer in three non-overlapping high-power fields (magnification x 200). The average area fraction per high power field per animal is presented.

### 2.6 Statistical analysis

GraphPad Prism v.5.01 (GraphPad Software, La Jolla, CA, USA) was used for statistical analysis. The data were tested for normal distribution with D’Agostino and Pearson omnibus normality tests. Normally distributed data was analyzed by one–way ANOVA with Tukey’s test for *post-hoc* analysis as appropriate. Non-parametric Kruskal Wallis tests followed by Dunn’s multiple comparison tests were used for data not normally distributed. All data are presented as mean standard error of mean (SEM) and significance was considered as p < 0.05.

## 3 Results

### 3.1 Physiologic data of the animals at birth

There were no significant differences in birth weights among the different study groups. All blood values in all groups were in the normal accepted range, the changes were not considered to be relevant (**Table 1**) (37). Sex was not considered to be relevant in terms of analysis as it does not influence the systemic inflammatory response (38).

**Table 1:** Physiologic data of the animals at birth: Values are presented as mean +/- SEM. * p < 0.05 versus control, § p < 0.05 versus 2d LPS.

	Animal Number	Sex Male/Female	Birth Weight (kg)	Cord Blood pH	Cord Blood pCO_2_ (mmHg)
Control	10	6/4	0.78 +/- 0.02	7.17 +/- 0.03	81.61 +/- 4.54
2d LPS	7	4/3	0.72 +/- 0.01	7.27 +/- 0.01*	65.81 +/- 1.67*
7d LPS	8	4/4	0.69 +/- 0.03	7.24 +/- 0.02	69.51 +/- 2.91
7d + 2d LPS	7	3/4	0.75 +/- 0.02	7.23 +/- 0.02	71.76 +/- 3.28
24d UP	7	3/4	0.69 +/- 0.02	7.14 +/- 0.02	84.5 +/- 4.17
24d UP + 2d LPS	8	3/5	0.76 +/- 0.02	7.17 +/- 0.01§	81.1 +/- 2.09§
24d UP + 7d LPS	7	1/6	0.73 +/- 0.01	7.19 +/- 0.02	77.04 +/- 2.90

### 3.2 Inflammatory cells in the fetal ileum

#### 3.2.1 T-lymphocytes (CD 3)

**Figure 2:**
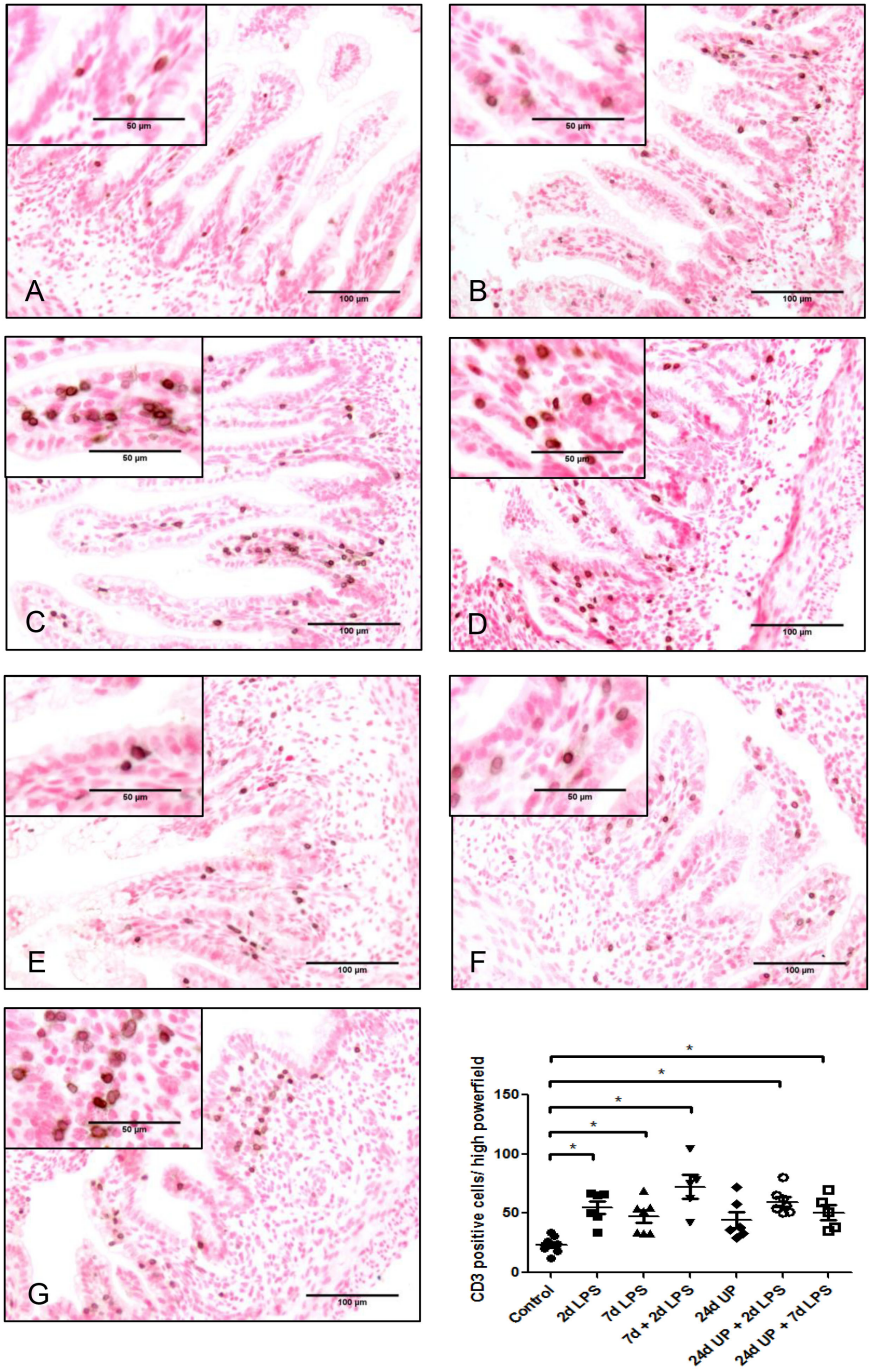
CD3 positive cells in mucosa and submucosal tissue of the fetal ileum. Counterstaining nuclear fast red 0.1%. Scale bar 100 µm in pictures and 50 µm in insets. **(A)**: control, **(B)**: 2d LPS, **(C)**: 7d LPS, **(D)**: 7d + 2d LPS, **(E)**: 24d UP, **(F)**: 24d UP + 2d LPS, **(G)**: 24d UP + 7d LPS. *p < 0.05 compared to control.

Only a few T-lymphocytes could be detected in the control group (**Figure 2**). The number of T-lymphocytes doubled in the 2d LPS study group and in the 7d LPS study group compared to the control group (p < 0.05) and tripled in number in the 7d + 2d LPS study group compared to control (p < 0.05). Exposure to 24d UP almost doubled the number of T-lymphocytes (but not reaching statistical significance), and exposure to 24d UP + 2d LPS as well as 24d UP + 7d LPS more than doubled the number of t-lymphocytes compared to control (p < 0.05).

#### 3.2.2 MPO positive cells

**Figure 3:**
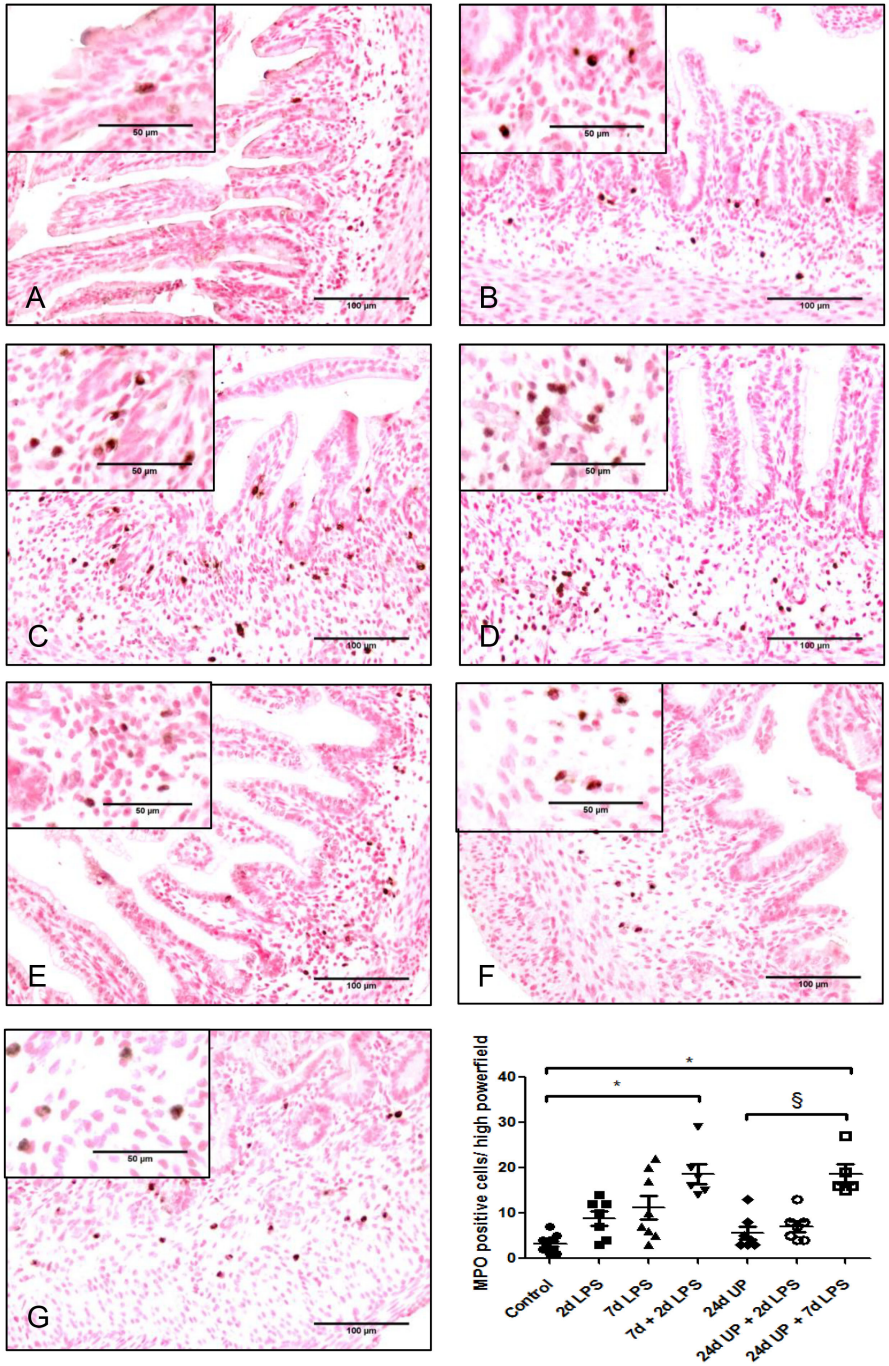
MPO positive cells in mucosa and submucosal tissue of the fetal ileum. Counterstaining nuclear fast red 0.1%. Scale bar 100 µm in pictures and 50 µm in insets. **(A)**: control, **(B)**: 2d LPS, **(C)**: 7d LPS, **(D)**: 7d + 2d LPS, **(E)**: 24d UP, **(F)**: 24d UP + 2d LPS, **(G)**: 24d UP + 7d LPS. *. p < 0.05 compared to control, § p < 0.05 compared to 24d UP.

Only a few MPO positive cells were detected in the control group whereas the number of MPO positive cells doubled after exposure to 2d LPS and tripled after exposure to 7d LPS, although these changes were not statistically significant (**Figure 3**). After exposure to 7d + 2d LPS the number of MPO positive cells was 5 times higher than the control group (p < 0.05). There were no significant changes after exposure to 24d UP or exposure to 24d UP + 2d LPS compared to control. The number of MPO positive cells was increased 6-fold higher after exposure to 24d UP + 7d LPS compared to control (p < 0.05). In between the different study groups there was a significant influx of MPO positive cells comparing exposure to 24d UP + 7d LPS to only exposure to 24d UP (p < 0.05). The number of MPO positive cells was 3-fold higher.

### 3.3 Changes in the enteric nervous system in the fetal ileum

#### 3.3.1 Synaptophysin

**Figure 4:**
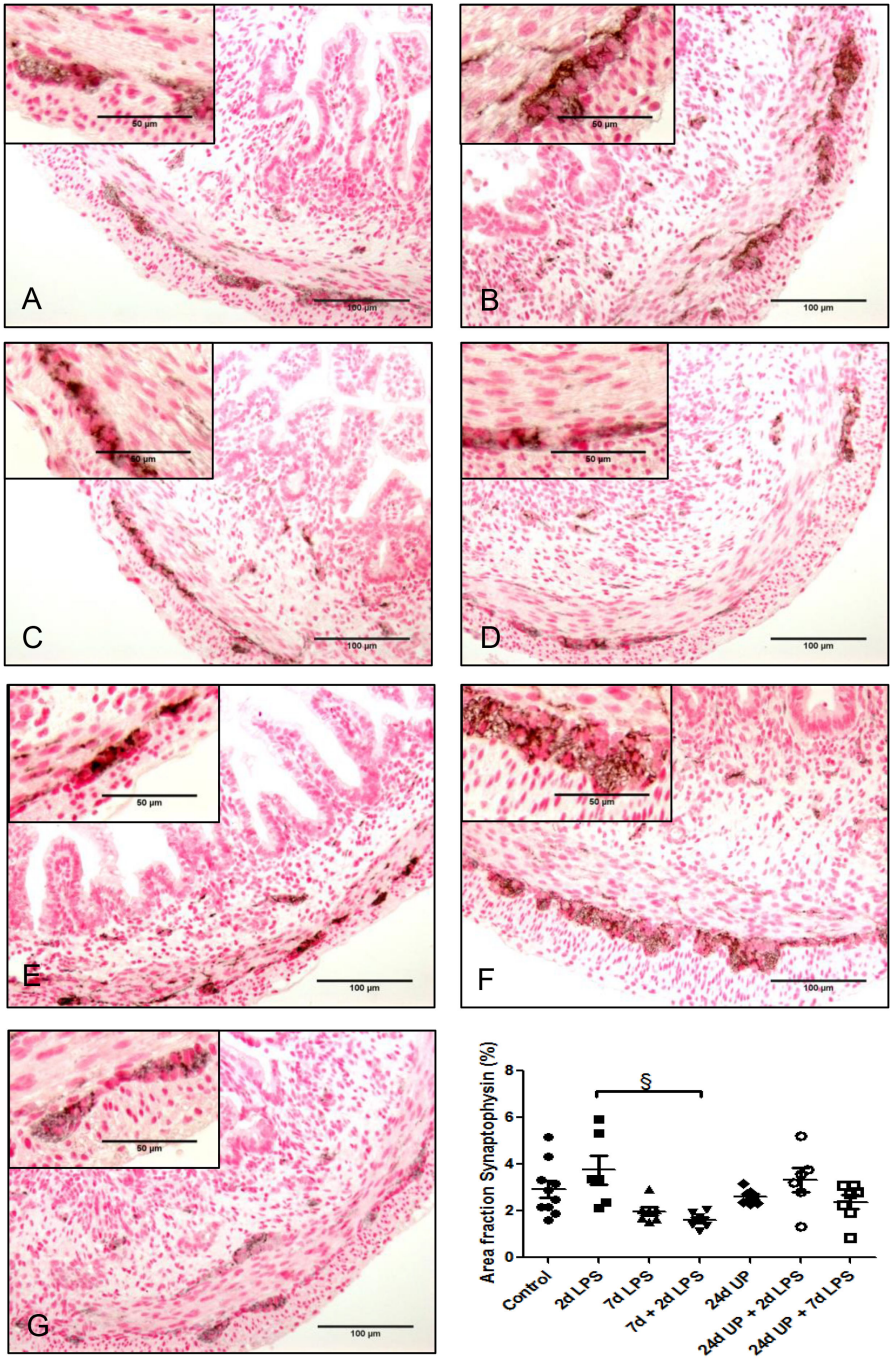
Synaptophysin in myenteric plexus of the fetal ileum. Counterstaining nuclear fast red 0.1%. Scale bar 100 µm in pictures and 50 µm in insets. **(A)**: control, **(B)**: 2d LPS, **(C)**: 7d LPS, **(D)**: 7d + 2d LPS, **(E)**: 24d UP, **(F)**: 24d UP + 2d LPS, **(G)**: 24d UP + 7d LPS. § p <. 0.05 compared to 2d LPS.

No significant changes in synaptophysin were demonstrated comparing the individual study groups with the control group (**Figure 4**). There was a significant reduction comparing exposure with 7d + 2d LPS to the 2d LPS study group (p < 0.05). UP exposure did not change synaptophysin expression.

#### 3.3.2 Enterochromaffin cells

**Figure 5:**
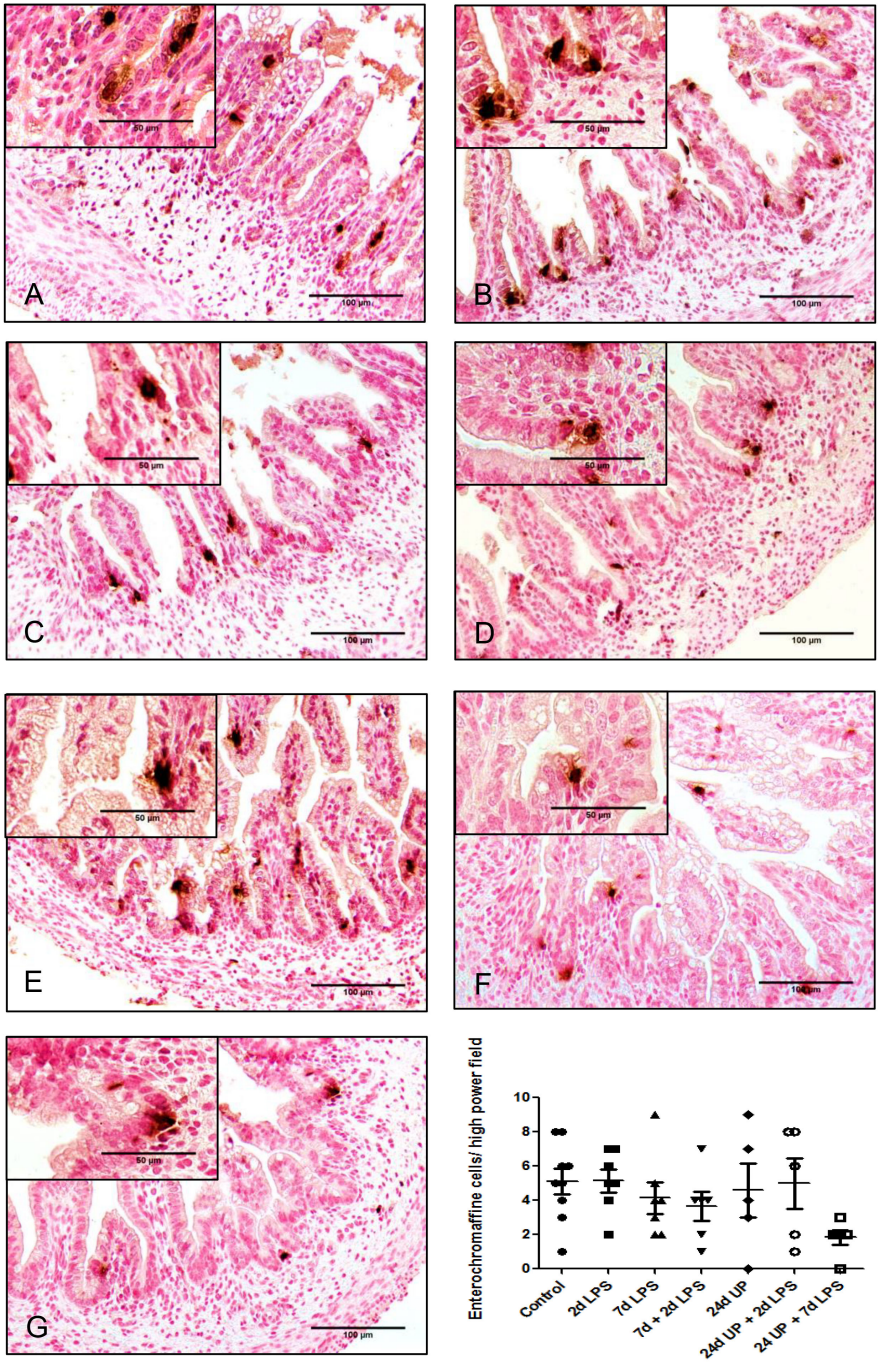
Enterochromaffine cells in mucosa of the fetal ileum. Counterstaining nuclear fast red 0.1%. Scale bar 100 µm in pictures and 50 µm in insets. **(A)**: control, **(B)**: 2d LPS, **(C)**: 7d LPS, **(D)**: 7d + 2d LPS, **(E)**: 24d UP, **(F)**: 24d UP + 2d LPS, **(G)**: 24d UP + 7d LPS.

No statistically significant changes in the number of enterochromaffin cells were detected between the different study groups compared to the control group (**Figure 5**). However, qualitatively the number of enterochromaffin cells was decreased to a third after exposure to 24d UP + 7d LPS compared to the control group.

#### 3.3.3 Neuronal nuclei

**Figure 6:**
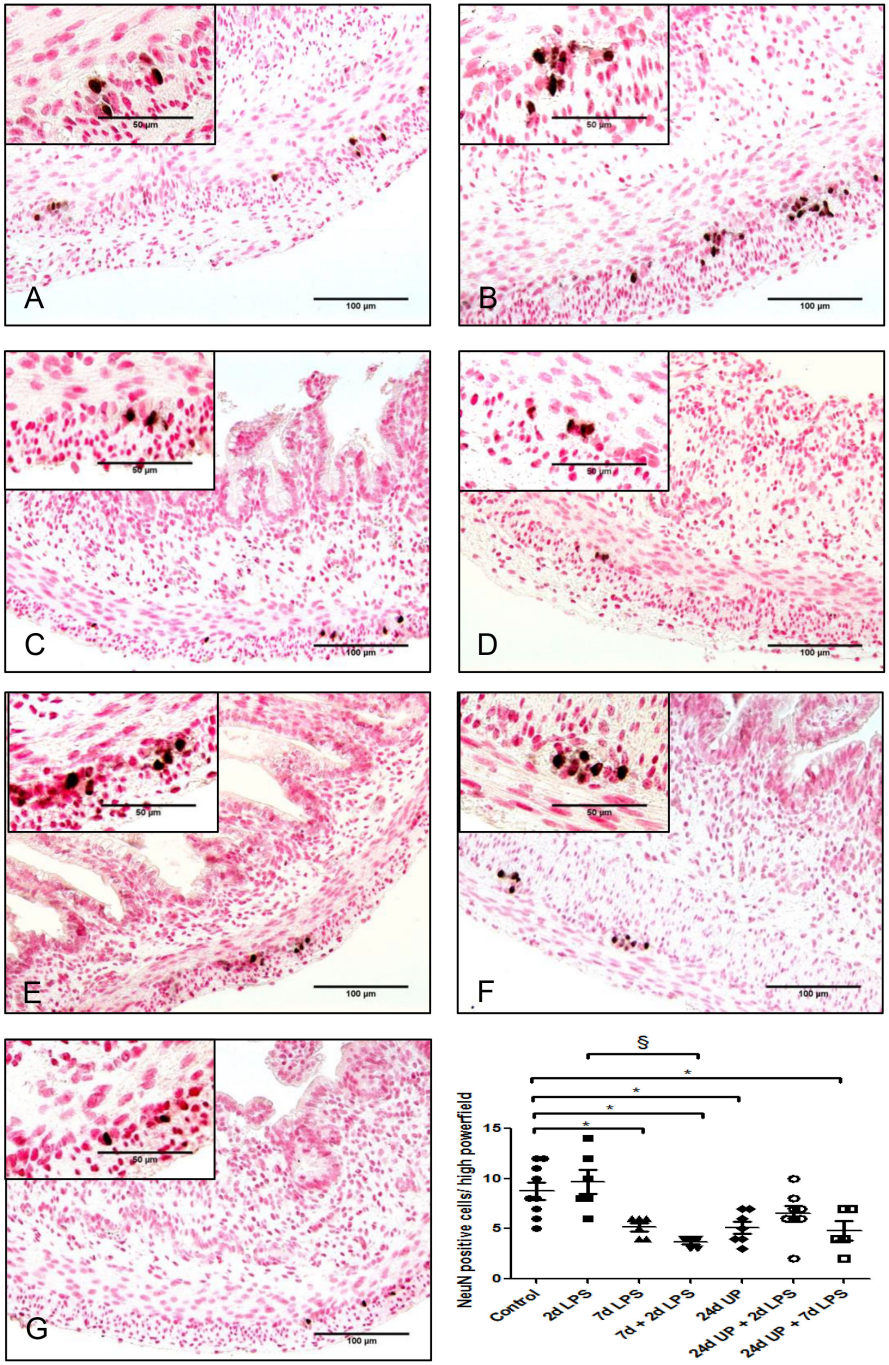
Neuronal nuclei in myenteric plexus of the fetal ileum. Counterstaining nuclear fast red 0.1%. Scale bar 100 µm in pictures and 50 µm in insets. **(A)**: control, **(B)**: 2d LPS, **(C)**: 7d LPS, **(D)**: 7d + 2d LPS, **(E)**: 24d UP, **(F)**: 24d UP + 2d LPS, **(G)**: 24d UP + 7d LPS. *p <. 0.05 compared to control, § p < 0.05 compared to 2d LPS.

There was a statistically significant reduction of nuclei of neurons after exposure 7d LPS compared to control (p < 0.05) as well as after the repeated exposure of 7d + 2d LPS compared to control (p < 0.05). Furthermore, there was a statistically significant reduction after exposure to 24d UP compared to control (p < 0.05) and after exposure to both 24d UP + 7d LPS compared to control (p < 0.05) (**Figure 6**). The reduction was the greatest with exposure to 7d + 2d LPS and for exposure to both 24d UP + 7d LPS as the number was decreased by half. Furthermore, there was a significant reduction of nuclei of neurons comparing exposure to 7d + 2d LPS to only 2d LPS (p < 0.05).

#### 3.3.4 Neuronal growth factor receptor

**Figure 7:**
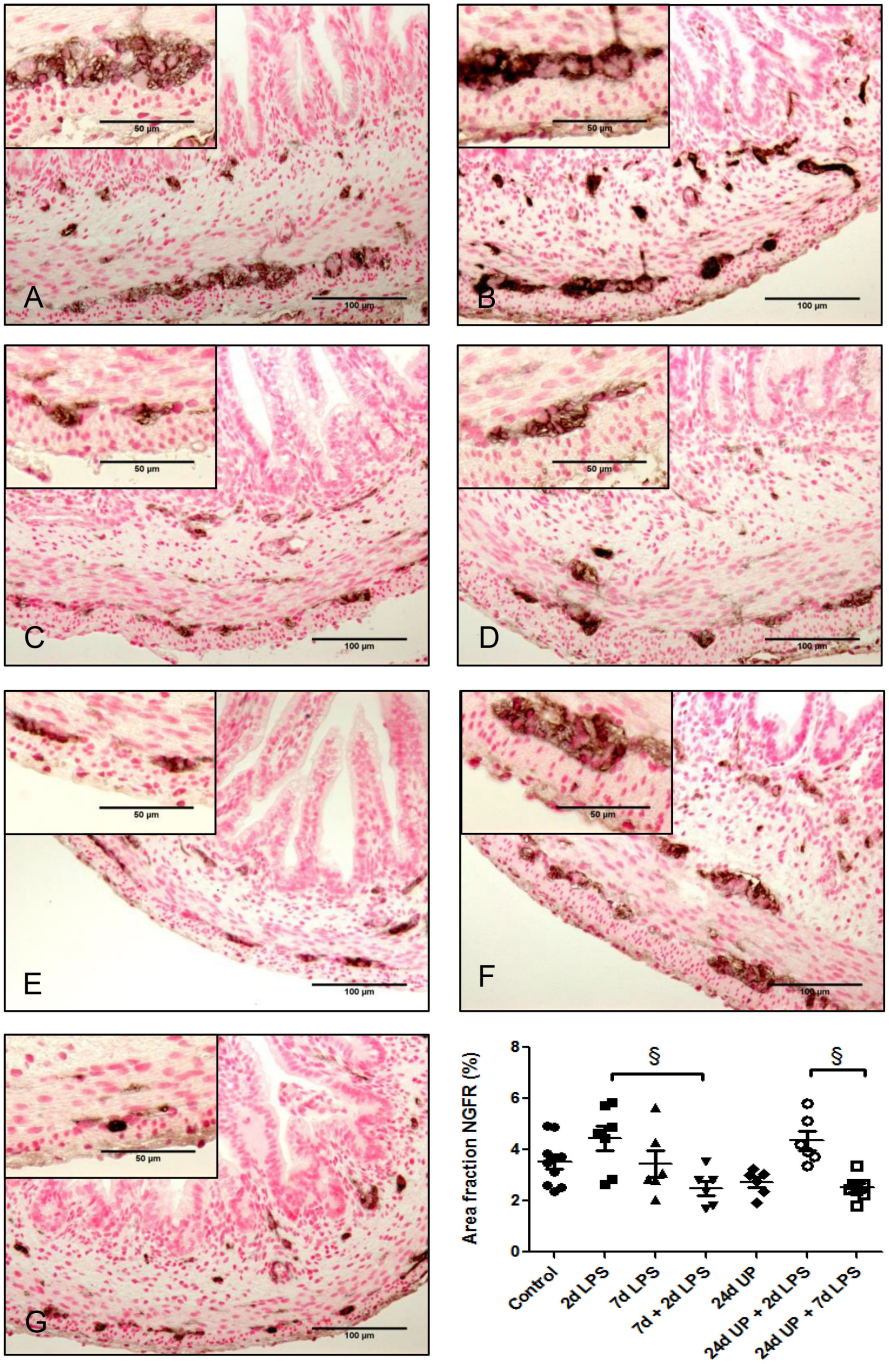
Nerve growth factor receptor positive area in myenteric plexus of the fetal ileum. Counterstaining nuclear fast red 0.1%. Scale bar 100 µm in pictures and 50 µm in insets. **(A)**: control, **(B)**: 2d LPS, **(C)**: 7d LPS, **(D)**: 7d + 2d LPS, **(E)**: 24d UP, **(F)**: 24d UP + 2d LPS, **(G)**: 24d UP. + 7d LPS. § p < 0.05 within the study groups.

No statistically significant changes were demonstrated comparing the different study groups with the control group (**Figure 7**). But there was a significant reduction comparing exposure to 7d + 2d LPS to 2d LPS (p < 0.05). Similarly, there was a significant reduction comparing exposure to 24d UP + 7d LPS to exposure to 24d UP + 2d LPS (p < 0.05). In both cases the area fraction was decreased to half.

#### 3.3.5 Vimentin

**Figure 8:**
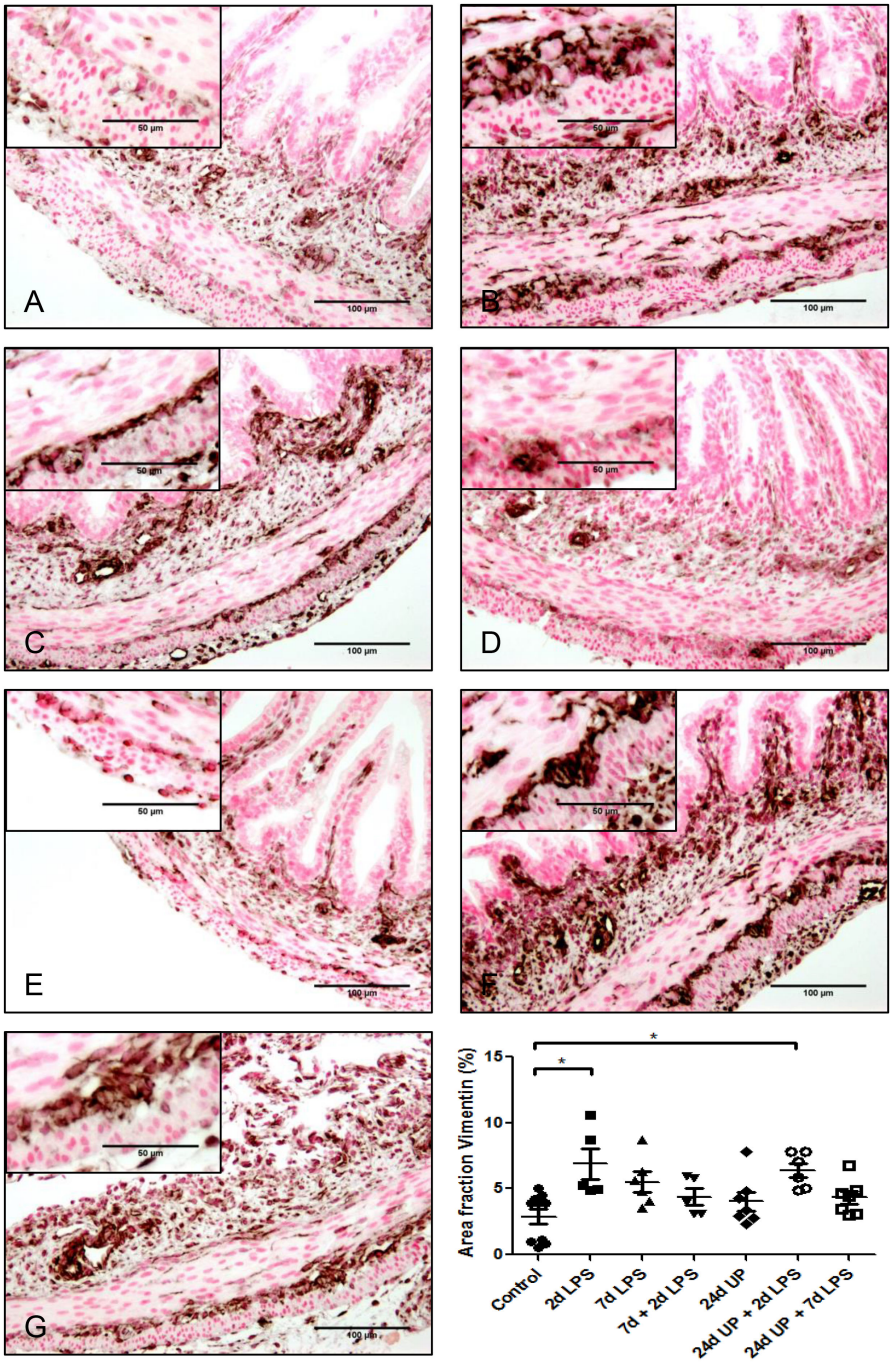
Vimentin positive cells (astrocyte like glial cells) in myenteric plexus of the fetal ileum. Counterstaining nuclear fast red 0.1%. Scale bar 100 µm in pictures and 50 µm in insets. **(A)**: control, **(B)**: 2d LPS, **(C)**: 7d LPS, **(D)**: 7d + 2d LPS, **(E)**: 24d UP, **(F)**: 24d UP + 2d LPS, **(G)**: 24d UP + 7d LPS. *p < 0.05 compared to control.

The area fraction of vimentin was twice as high after exposure to 2d LPS compared to control (p < 0.05) as well as after exposure to 24d UP + 2d LPS compared to control (p < 0.05) (**Figure 8**). In all other groups the area fraction of Vimentin quantitatively increased but not significantly compared to the control.

### 3.4 Summary

To make a brief summary, significant fetal ileum changes compared to the control group were as followed:

When being exposed to only LPS 2 days before delivery the increase in CD3 positive T-lymphocytes and in the area fraction of vimentin were significant.

When being exposed to only LPS 7 days before delivery the increase in CD3 positive T-lymphocytes and the decrease in neuronal nuclei were significant.

When being exposed to LPS 7 and 2 days before delivery the increase in CD3 positive T-lymphocytes and MPO positive cells and the decrease in neuronal nuclei were significant.

When being exposed to only UP 24 days before delivery the decrease in neuronal nuclei was significant.

When being exposed to UP 24 days and LPS 2 days before delivery the increase in CD3 positive T-lymphocytes and in the area fraction of vimentin were significant.

When being exposed to UP 24 days and LPS 7 days before delivery the increase in CD3 positive T-lymphocytes and MPO positive cells and the decrease in neuronal nuclei were significant.

Significant differences comparing the different study groups to each other are not listed.

All results are summarized in **Table 2**.

**Table 2:** Summary of the results: Values are presented as mean +/- SEM.

	CD3	MPO	Synaptophysin	Enterochromaffine cells	NeuN	NGFR	Vimentin
2d LPS	**↑**						↑
7d LPS	↑		n		↓		
7d + 2d LPS	↑	↑	↓ (2d LPS)		↓+ (2d LPS)	↓ (2d LPS)	
24d UP					↓		
24d UP + 2d LPS	↑						↑
24d UP + 7d LPS	↑	↑+ (24d UP)		.	↓	↓ (24d UP + 2d LPS)	

↑ significant increase in cell number or area fraction compared to control (p < 0.05), ↑ (study group) significant increase in cell number or area fraction compared to study group (p < 0.05), ↑ + (study group) significant increase in cell number or area fraction compared to control + study group (p < 0.05). ↓ significant decrease in cell number or area fraction compared to control (p < 0.05), ↓ (study group) significant decrease in cell number or area fraction compared to study group (p < 0.05), ↓ + (study group) significant decrease in cell number or area fraction compared to control + study group (p < 0.05). No significance when nothing is indicated.

## 4 Discussion

The incidence of histologic chorioamnionitis is inversely correlated with gestational age at preterm birth (1, 5) and the risk of NEC as a complication of prematurity is increased by chorioamnionitis/funisitis (10). We therefore assessed whether exposure to intra-uterine inflammatory stimuli resulted in intestinal inflammation and changes in the ENS at extremely low gestational age (i.e., 60% of term). In a previous study at 80% of term gestation, we found that repetitive microbial exposure did not further increase injury of the ileum. Chronic intra-amniotic UP exposure caused significant structural ENS alterations, whereas these changes were not found after re-exposure of chronic UP-exposed fetuses to LPS for 2 or 7 days (17). Furthermore, chorioamnionitis is often caused by multiple pathogens (6). Tolerance type mechanisms for repeated exposure to inflammatory exposures were described previously (21, 23, 24) for the lungs and systemically for more mature fetal sheep (17). We therefore assessed a multiple hit model of chorioamnionitis with respect to the intestine of extremely premature ovine fetuses to test for tolerance at 60% of term gestation (**Table 2**).

The extremely immature ovine fetuses at 94d gestation (term = 150d) had both innate and acquired immune system inflammatory responses in the ileum to chorioamnionitis induced by LPS. The number of inflammatory cells was increased after two exposures to LPS. Hence, even at this young gestational age components of the innate and acquired immune system are already functional. In fetal sheep at 120-123 days of gestational age after repetitive LPS injections into the amniotic fluid, a cross tolerance to several toll like receptor agonists in blood and lung inflammatory cells has been shown (21, 22). In this study we could not detect a similar phenomenon in the ileum. The difference may be attributed to a different gestational age as well as a different organ. In addition, the processing of inflammatory signals from amniotic fluid in lung and gut may be different at this gestational age.

We have furthermore shown that the timing is important for the immune reponse (39). Previous UP exposure followed by an LPS challenge increased the inflammatory response. Tolerance mechanisms as described previously (23, 24) could not be detected. Similar to our findings on the gut a more severe pulmonary inflammation has also been demonstrated after chronic UP and subsequent LPS exposure at this young age in the lung (26). We take this as another hint that tolerance mechanisms may be gestational age dependent (17, 21, 23, 24).

Furthermore, double exposure to LPS *in utero* or combined exposure to UP and subsequent exposure to LPS caused structural changes in the myenteric plexus, which is representative for the ENS. Consequences were more pronounced after a longer and after multiple exposures to inflammatory stimuli in general. Interestingly, the number of inflammatory cells was associated with the structural changes such as a decrease in neuronal nuclei in the ENS for example.

There have been no evaluations for structural changes of the ENS after chorioamnionitis. We evaluated the negative effects of chorioamnionitis, induced by UP and/or LPS, on the ENS at this very early gestational age, showing mainly that there is a loss in neurons. We speculate that these findings (loss of neurons), can be extrapolated to preterm babies which may help to understand some clinical findings. To be more precise one can assume that feeding intolerance is for example derived from a most probably restricted function of the ENS when there is a structural loss of neurons.

The enteric nervous system is responsible for muscular activity in the gut as well as for fluid secretion of the secretory epithelial cells (15). Both mechanisms are important for the frequency of stool. In a recent study the first meconium elimination in preterm infants, who later on developed NEC, was significantly delayed and subsequent frequency of stools was significantly lower compared to control (40). We demonstrated structural changes of the ENS after chorioamnionitis induced by UP and/or LPS, which may indicate functional loss and the association of funisitis and necrotizing enterocolitis in clinical studies (10). The role of the endothelium in the progression from chorioamnionitis to funisitis warrants further studies. The “vascular hypothesis” of inflammatory/infectious diseases may be the explanation for many clinical associations which mandates further evaluation.

The ENS participates in the maintenance of the intestinal microbiome (15). Normally, intestinal colonization starts during birth when the newborn contacts the mothers’ vaginal flora (41, 42). When being exposed to chorioamnionitis the fetus contacts commensals and pathogens earlier. We therefore assume that there is an abnormal colonization of the intestine before being born irrespective of birth mode (43). If the ENS is compromised, the maintenance of a healthy luminal microbiome may be even more complicated. This could be another reason why funisitis is associated with a higher risk of developing NEC (10). This hypothesis warrants experimental proof.

The adverse effects of chorioamnionitis on the gut are not limited to the newborn period. Young adults who recovered from NEC had lower pain thresholds and pain tolerance compared to controls (13). NEC is a disease involving local as well as systemic inflammation and necrosis leading to visceral pain. Again the ENS with its 50.000 extrinsic and over 100 million intrinsic afferent neurons (44) is essential for pain perception.

Subsequent research will have to test if the structural changes, we have demonstrated, come along with functional loss and hence with different pain tolerance as well as a different pain threshold as a marker of the affected neuronal input from the ENS.

Our study has several limitations. We have only analyzed structural changes of the ENS after exposure to chorioamnionitis induced by UP and/or LPS. Therefore, no data on the functioning of the ENS is available. In addition, we have only assessed consequences of chorioamnionitis concerning the extreme immature ileum but not analyzed any other parts of the gut. We have not studied long term effects either. Moreover, the introduction of pro-inflammatory/infectious agents into the amniotic fluid is a reductionist approach to mimic a complex clinical entity with high variability.

In conclusion, we have demonstrated that after longer and after multiple exposures to pro-inflammatory stimuli inflammatory responses as well as structural changes of the ENS are more severe at extremely low gestational age. Consequently, a new insight into mechanistic correlations between chorioamnionitis, inflammatory and structural changes in the gut and in the enteric nervous system warrant further studies to determine the clinical importance of these changes.

## Data availability statement

The raw data supporting the conclusions of this article will be made available by the authors, without undue reservation.

## Ethics statement

The animal study was reviewed and approved by University of Western Australia, Subiaco, Western Australia, Australia, with approval of the Animal Ethics Committee (reference number RA/3/100/312).

## Author contributions

JH, DS, BK: Concept of the study, analysis of data. JH: Execution of experiments, data acquisition, analysis, writing of manuscript. MK, OS, JN, SK, AJ, BK: Design, excecution of animal experiments. OS: Preparation and handling of bacterial inoculation. JH, MK, OS, DS, JN, SK, AJ, BK: Reviewing of manuscript. All authors contributed to the article and approved the submitted version.
